# Embryonic Circadian Rhythm Establishment, Homeostasis, and Dysfunction During Organogenesis

**DOI:** 10.1155/sci/9621027

**Published:** 2025-09-18

**Authors:** Si-Lin Chen, Hang Zhou, Yu-Mei Li, Yun-Wen Zheng

**Affiliations:** ^1^Institute of Regenerative Medicine, and Department of Dermatology, Affiliated Hospital of Jiangsu University, Zhenjiang 212001, Jiangsu, China; ^2^Guangdong Provincial Key Laboratory of Large Animal Models for Biomedicine, and South China Institute of Large Animal Models for Biomedicine, School of Pharmacy and Food Engineering, Wuyi University, Jiangmen, Guangdong 529020, China; ^3^Haihe Laboratory of Cell Ecosystem, Institute of Hematology, Chinese Academy of Medical Sciences, Tianjin 300020, China; ^4^Department of Medical and Life Sciences, Faculty of Pharmaceutical Sciences, Tokyo University of Science, Tokyo 125-8585, Japan; ^5^International Research Center for Medical Sciences, Kumamoto University, Kumamoto 860-0811, Japan

**Keywords:** circadian rhythms, clock genes, dysfunction, embryo, homeostasis

## Abstract

**Background:** Circadian rhythms play a crucial role in the management of the temporal organization of various physiological and cellular processes in mammalian cell types. These rhythms are involved in the regulation of the cell cycle and metabolism and have implications for pathogenesis and physiological homeostasis. Therapeutic approaches that target circadian regulation are emerging for the treatment of digestive disorders, metabolic diseases, and cancer. The proper coordination of cellular clocks is essential for tissue homeostasis and metabolic health.

**Methods:** The exact mechanisms governing the development and regulation of the circadian clock during embryo development are still unclear. However, embryo rhythms, such as those in the suprachiasmatic nucleus (SCN), liver, kidney, adrenal gland, and intestinal system, are believed to be influenced by maternal rhythms during different stages of embryo development. These rhythms then oscillate independently from the timing marked as the key to embryo development. In this review, we synthesize our laboratory experience and summarize current research to provide insight into how circadian rhythms regulate and synchronize organ functions for growth and differentiation during embryo development.

**Results:** Our laboratory experience and current research suggest that circadian rhythms are involved in the regulation of organ functions during embryo development. Maternal rhythms may entrain embryo rhythms during specific developmental stages, leading to independent oscillation and coordination of organ functions. This knowledge has implications for regenerative medicine and potential clinical applications.

**Conclusions:** Circadian rhythms play a crucial role in the coordination and synchronization of organ functions for growth and differentiation during embryo development. Understanding the regulation of circadian rhythms in embryos can provide valuable information for regenerative medicine and potential clinical applications. More research is needed to fully unravel the mechanisms underlying circadian clock development and regulation during embryo development.

## 1. Introduction

Circadian rhythms refer to the physiological processes and behaviors that follow a daily cycle, including sleep-wake cycles, hormone secretion, and regulation of body temperature. These rhythms are governed by the body's internal biological clock, which is synchronized by natural cues such as light and darkness. Disruptions in circadian rhythm can have negative impacts on health and well-being, including an increased risk of sleep disorders, mood disorders, and metabolic dysfunction. The suprachiasmatic nucleus (SCN), located bilaterally in the anterior hypothalamus, functions to integrate photic inputs and synchronize environmental cues for survival [1]. As the central pacemaker of the mammalian circadian system, it largely governs the rhythms of peripheral clocks through intrinsic regulatory mechanisms [2]. The cell-autonomous molecular circadian clock, the transcription-translation feedback loop (TTFL), is highly conserved. CLOCK-Bmal1 activates target gene transcription, while negative PER and CRY protein families provide negative feedback to control their own transcription [3]. iPSC reprograming efficiency were also affected by the CRY1, reflects the important role of clock genes in early development of life [4]. At the molecular level, the circadian timekeeping mechanism is rooted in these intricate feedback loops between a set of clock genes, comprising Bmal1, PER1, PER 2, PER 3, CRY1, CRY 2, and CLOCK, which orchestrate downstream gene expression and modulate diverse cellular activities [5].

Circadian rhythms play a key role in the regulation of numerous physiological processes in mammals, including those that govern cardiovascular, metabolic, and immune function [6]. Despite this, the precise nature and influence of the circadian system on embryo development remain relatively unclear. Although there exist experimental data suggesting the existence of clock mechanisms prior to birth, knowledge regarding the function of gestational clocks remains inconsistent. Furthermore, little is known about the extent to which embryo rhythms depend on maternal and environmental signals or the role of circadian oscillations in embryo development [7]. Frontier scientists are particularly interested in understanding how stable circadian rhythms develop in various peripheral systems, subsequently regulating the organism's developmental maturation. Recent investigations of the regulation of embryo development by the circadian system have revealed the pivotal role of individual clock genes in this process [8]. Various studies have also explored the regulatory roles of different circadian genes in the gastrointestinal and adrenal systems [9, 10].

Numerous studies have shown that the biological clock acts as a critical developmental timer in ontogeny, regulating organogenesis. During early development, segmentation profoundly impacts the overall developmental process, with CLOCK/Bmal1-mediated transcriptional regulation at the forefront [11]. Bmal1, one of the core clock genes, is essential for maintaining circadian rhythm function, as evidenced by the severe loss of function observed in Bmal1 deficient mice [12]. Furthermore, Bmal1 serves as a vital regulator of gastrulation and multicellular differentiation, highlighting the indispensable role of circadian rhythm regulation in embryo development [13]. However, premature expression of Bmal1 can also alter circadian clock oscillation and cellular function. In a recent study by Yang et al. [14], conditional Bmal1 knockout mice during adulthood exhibited improved atherosclerosis and hair growth, while losing Bmal1 characteristics. These findings suggest that Bmal1 knockout, particularly during embryo development and before maturation, disrupts the circadian rhythm, with implications beyond the maintenance of the 24 h oscillation. The unconventional effects of the circadian gene impact health, aging, and life expectancy.

However, there is limited understanding of the interaction between chronobiology and developmental biology, particularly with respect to the influence of circadian rhythms on embryo and organ development, which is the main focus of the present review. Timing, initiation, and degree of circadian rhythm synchronization often leads to harmonization of organ function, promoting individual growth and development, provoking scholarly interest.

## 2. Circadian Clock Matters in Embryos

Recent studies have highlighted the crucial role of clock gene oscillations in successful pregnancy and delivery, while also indicating that circadian disturbances caused by shift employment, nighttime light pollution [15], and other factors can alter ovarian and placental function [16–19]. Circadian modulation could improve the function of hPSC-derived tissues [20]. In mammals, maternal signals transmitted through the placental system are vital for the maturation and synchronization of the fetal circadian rhythm system [21]. While breast milk provides rhythmic maternal signals in the early postpartum period until the newborn SCN and the circadian rhythm system mature [22]. Thus, the maternal circadian rhythm may play a crucial role in this cell-fate symphony.

According to some researchers, embryos possess an autonomous circadian rhythm. Studies have indicated that 43% of protein-coding genes exhibit circadian rhythms, reaffirming the vital role circadian rhythms play in physiological activities. The presence of light during the early life of *Drosophila melanogaster* synchronizes the circadian clock, supports brain development, and influencing adult behavior [23]. Circadian genes typically produce tissue-specific impacts. The peripheral clock can be scaled, coordinated, and separated from the SCN through nonphotic cues when oscillated in vitro. These genes are active in different systems, with inherent rhythmic coordination, shedding light on the regulation of rhythmic development in various organs. Landgraf et al. [7] demonstrated that there was an intrinsic circadian clock in the SCN, liver, and kidney of developing mice in ED13, remaining relatively stable throughout embryogenesis and postpartum. Circadian rhythms of the embryo can develop without maternal or other environmental time signals, and disrupting maternal and fetal rhythms can affect development [7]. Ameneiro et al. [24] identified the circadian rhythm generated in embryos through in vitro bioluminescence imaging, detecting a circadian period developed in PER2 at ED14.5.

Scholars suggest that the role of the mother is closely associated with embryo rhythm formation, indicating that independent establishment of circadian rhythms during the embryo period may not be possible due to differential maturation of circadian rhythms in the SCN and pars tuberalis (PT). It has been observed that, despite the significant role of circadian genes in embryo development, robust autonomous rhythmic oscillation cannot be established in the embryo. The reason is complicated, while a study indicate that CLOCK protein is essential for the regular circadian rhythms, due to the CLOCK mRNA translation have been restrained by the Dicer/Dgcr8-mediated posttranscriptional suppression, the core circadian TTFLs are not established in the early stage of embryonic development [25]. No interspecific differences were observed in the expression profiles of circadian genes in oocytes and preimplantation embryos of diurnal and nocturnal species (i.e., cattle and rabbits), respectively [26]. Cao et al. [8] found that circadian expression of Bmal1, CLOCK, and Per1 mRNA in the maternal heart, liver, kidney, and quadriceps femoris during embryo development is related to cell development and differentiation, although circadian rhythms cannot be established in embryos. This lack of functional circadian rhythms is not due to deletion of the core circadian genes [27], such as Bmal1, which can even be significantly downregulated in synchronously stimulated hiPSCs [28]. Although no circadian oscillations were observed in mouse embryonic stem (ES) cell cultures [29], human ES cells [30], and hiPSCs [31], circadian genes are active in ES cells [32] and influence differentiation potential in metabolic control that determines cell fate [24]. The intrinsic rhythmicity of the SCN cell population has not yet developed before E18-E19, with the observed rhythms in this fetal stage derived from maternal behavior [33]. Therefore, critical time points that involve biological processes, such as regulation of specific gene networks, activation, and inhibition, may influence the exchange of rhythmic dominance of the embryo and dynamic changes in gene expression during embryo development. The significant influence of the circadian rhythm system and circadian genes on embryo development is recognized and prompts further exploration.

## 3. The Expression and Influence of Circadian Rhythm in Embryos

In recent years, there has been an increasing consensus on the circadian rhythms of embryos [34]. As embryo development is closely related to the mother, investigating the circadian rhythm of the embryo requires studying the relationship between the circadian rhythms of the mother and the embryo. Researchers have explored this relationship, which involves a complex and dynamic developmental process that prepares the embryo for postbirth living conditions. In different organs, maternal signals can drive the clock of the fetus and the newborn, and during this process there is a meaningful interaction between the mother and the fetus.  Table 1 highlights some important embryonic stages at which embryonic organs begin to oscillate.

PT located in the anterior pituitary, is a peripheral oscillator that is heavily relying on rhythmic melatonin signals. In adults, PT is the primary target of melatonin [41]. The circadian protein rhythm in fetal PT may depend on maternal melatonin signaling [35]. Therefore, maternal melatonin is crucial for the establishment and maintenance of the circadian rhythm of peripheral fetal peripheral oscillators [39, 42–44]. Melatonin produced from the pineal gland, while the pineal glands are unmature in fetal rodent spices, which cannot produce melatonin, suggesting fetal clock oscillation were affected by maternal melatonin secreted from the placenta [25, 42]. Maternal melatonin supplies the fetus with photoperiodic signals and consistent rhythm, bringing about rhythmic harmony that coordinates the physiological functions of the mother, placenta, and fetus [45]. Clinical evidence shows that melatonin deficiency could cause life-threatening conditions in both the mother and the fetus [46]. It is also a vital factor for the evaluation of fetal risks and the timely prevention of endocrine disorders, such as type 2 diabetes, in offspring [47]. Furthermore, maternal pineal melatonin deficiency could determine abnormal brain programing of the offspring. A study on maternal melatonin deprivation (MMD) during pregnancy and lactation showed that it delayed the development of physical characteristics, neural development, and cognition in male offspring, calling for public health attention for mothers who work night shifts [48]. Disruption of the intestinal circadian rhythm has been linked to various consequences, such as diarrhea [49], ulcers [50], inflammatory bowel diseases [51, 52], and colorectal cancer [53]. It has also been suggested that rhythm disorder could be the initial manifestation of inflammatory bowel diseases [54]. Researchers have found that the amplitude of individual circadian gene expression profiles changes with age, between the 20th day of the rat embryo (ED20) and the 30th day after birth (P30), especially for Per2, Rev-erb, and Bmal1 in intestinal circadian rhythm. Epidemiological research demonstrates that adverse events during the embryonic stage can affect the development of the fetal kidney, thus increasing the incidence of hypertension and chronic kidney disease. Therefore, optimizing maternal health and early childhood nutrition can slow this trajectory, thus mitigating the global prevalence of hypertension and kidney diseases [55]. Normal kidney development requires an intact fetal clock to establish the structure and function of adult organs. Landgraf et al. [7] contend that the kidney of mice exhibits an endogenous circadian clock at E13. Some investigators demonstrate that Per2 shows a distinct circadian rhythm at ED18.5 [38], while others maintain that at ED20, CLOCK, Per2, Rev-erb, ENaC, Sgk1, Nhe3, and Avprr2 evince internal, autonomous oscillation at the molecular level [37]. However, it is universally recognized that these rhythms arise due to maternal circadian rhythm signals, such as fluctuating maternal circadian rhythm endocrine levels [56] and regular food ingestion [57]. Undeniably, circadian rhythm regulation plays a crucial role in embryo development and maturation, especially for interdependent organs. In adults, plasma glucocorticoid serves as a systemic hormone signal that exhibits changes in circadian rhythm and profoundly affects various aspects of psychology and physiology [58, 59]. The fetal adrenal gland, which is a powerful peripheral clock, is known to be regulated by maternal melatonin, as evidenced by recent studies [60]. Administering glucocorticoid treatment to pregnant women has been found to reduce the risk of behavioral disorders associated with circadian rhythms [61]. In the rat fetal adrenal gland, circadian genes such as Bmal1 and Per2 exhibit strong periodic oscillations, as does the acute regulatory steroid protein (StAR), the Mt1 melatonin receptor, and the early growth response protein 1 (Egr-1) [35]. However, chronic photoperiod shift during pregnancy, also known as gestational chronodisruption, can have negative effects on adult and fetal adrenal biorhythms and function, potentially leading to metabolic adaptation, increased risk of chronic diseases [62], and an increased incidence of benign and malignant tumors [63]. As a circadian signal, glucocorticoids aid in the regulation of circadian genes, which may be the key to understanding the disease and the advancement of clinical applications of glucocorticoids. In hepatology, glucocorticoids are recognized for their ability to control the transcription of crucial enzymes involved in glucose and lipid metabolism. However, its use in various clinical practices, such as Addison's disease, Cushing's syndrome, and long-term clinical use, could pose serious metabolic challenges to vital metabolic organs, like the liver [64]. It was discovered that there already existed endogenous self-sustained oscillations in mice livers at ED13 [7]. Studies have also detected that chronic photoperiodic changes during pregnancy lead to a decrease in liver transcription levels of Bmal1 in P90, while PAI-1 is upregulated, impacting the expression of the liver gene of adult male offspring and increasing their risk of developing cardiovascular diseases [65]. Similarly, single cell sequencing has established a relationship between the circadian rhythm regulator BHLHE40 during liver development, liver metabolic behavior, and liver cell differentiation. This analysis of circadian rhythms during liver development provides valuable information for further study of liver metabolism, development, and diseases [66].

## 4. Limitations and Challenges

### 4.1. Precise Location for Circadian Development

Current research on circadian clocks in embryos typically uses whole tissues to assess gene oscillations [37], which precludes examination of circadian rhythm regulation in specific regions of organs. Although bioluminescence imaging has been used in vitro to investigate potential sites of rhythm generation in the SCN [40] and kidneys [38], further exploration is required to fully elucidate the complexities of rhythmic development in peripheral systems and pinpoint precise functional locations. Tissue microdissection technology may facilitate the study of oscillatory gene activity in specific regions in future research [67]. Expanded sample sizes and more granular time scales are also essential, as is comparative analysis with different sexes.

### 4.2. Intercircadian Communication

In addition to the traditionally recognized central circadian system that guides peripheral systems, peripheral systems may also be able to affect the central circadian system. One such example of intercircadian communication is skin, which can function as an organ for the secretion of β-endorphins [68] and corticotropin-releasing hormone (CRH), hormones that can affect the hypothalamic–pituitary–adrenal (HPA) axis [69]. Studies have shown that exposure to light can rapidly activate neuroendocrine effects, resulting in increase in plasma levels of pituitary-independent neuropeptides and corticosterone, leading to significant immunosuppression [70]. The interplay between skin and brain facilitates the effective role of skin as a potential mediator in the adaptive response to changes in the external environment. Therefore, we are also encouraged to explore in developmental biology whether this distinct potential correlation exists prenatally.

### 4.3. Applications Prospects in Comparable and Robust Models

Researchers use various models, and experimental conditions vary according to the specific study. For example, the maintenance of pregnant rats and the sampling methods are different. Some researchers kept rats in the dark before sampling [71], while others sampled rats during the light/dark cycle [72]. This methodological discrepancy prompts inquiries into the comparability of research findings. Efforts are underway to improve the situation [73]. Embryos cannot be observed in the mother's womb until they reach a certain size that can be detected by ultrasound, usually around 3 weeks after conception. To study human development without using real embryos, researchers rely on stem cells that can be guided to form in vitro embryo-like structures. Human organoids that can be coaxed from ES cells, which have become popular instruments for studying embryo circadian rhythm because they simulate human genes and avoid ethical issues, are complex multicellular in vitro systems that do not have systemic signaling factors, making them an attractive mechanism for assessing the endogenous tissue-specific functions of peripheral circadian rhythms. These structures contribute to an expanding range of embryo models that help to understand the processes that lead to early pregnancy loss. This area of study is crucial, as approximately 60% of human pregnancies are reported to end within the first 14 days [74]. The goal is to help researchers improve reproductive technologies, mitigate miscarriages, and treat congenital diseases.

### 4.4. Mining of Unconventional Functions

The influence of circadian genes and rhythms extends beyond their auxiliary functions, as they have been found to affect various biological processes. Specifically, circadian rhythms have been implicated in embryo formation and fetal development, highlighting their potential clinical relevance in preventing pregnancy-related diseases [75]. Furthermore, the intersection of calorie restriction and diurnal regulation has been shown to improve the longevity of mice, indicating the intricate relationship between these systems [76]. Furthermore, increasing evidence suggests that disruption of circadian rhythms is associated with a variety of pathologies, such as obesity, type 2 diabetes, and nonalcoholic fatty liver disease (NAFLD) [77]. The use of ES cells for cell differentiation and reprograming provides a unique window to observe the process of circadian rhythm formation and its association with stem cell aging, and may provide novel insights into understanding the molecular organization of circadian rhythm in mammals [29]. Studies have shown that they are able to target the alleviation of aging-associated syndromes [78]. By integrating cell culture techniques and chronobiology, the potential for an increase in the number of viable cells and unexpected results during growth and differentiation is promising. In summary, the impact of circadian genes and rhythms is far-reaching and may have implications for understanding and treating various diseases.

### 4.5. Countermeasures for Oncology Based on Chronobiology

Research on circadian rhythms has resulted in the development of a new time-based treatment strategy and prescription [79], while investigating tissue-specific embryo rhythms has expanded the possibilities in the field of chronobiology. These protein-coding genes can also be targeted for drug development, even if their sole function is drug transport or metabolism, providing a new avenue for precision medicine [80]. Circadian dysregulation can drive cancer progression, leading to the emergence of chronochemotherapy as a promising therapeutic option [81]. By enhancing the regulation of our rhythmic genes from the beginning, we may enjoy a more harmonious, fluid, and enriched symphony of life, extrapolating from the end of our life ensemble and considering the beginning.

## 5. Conclusions and Future Perspectives

**Figure 1:**
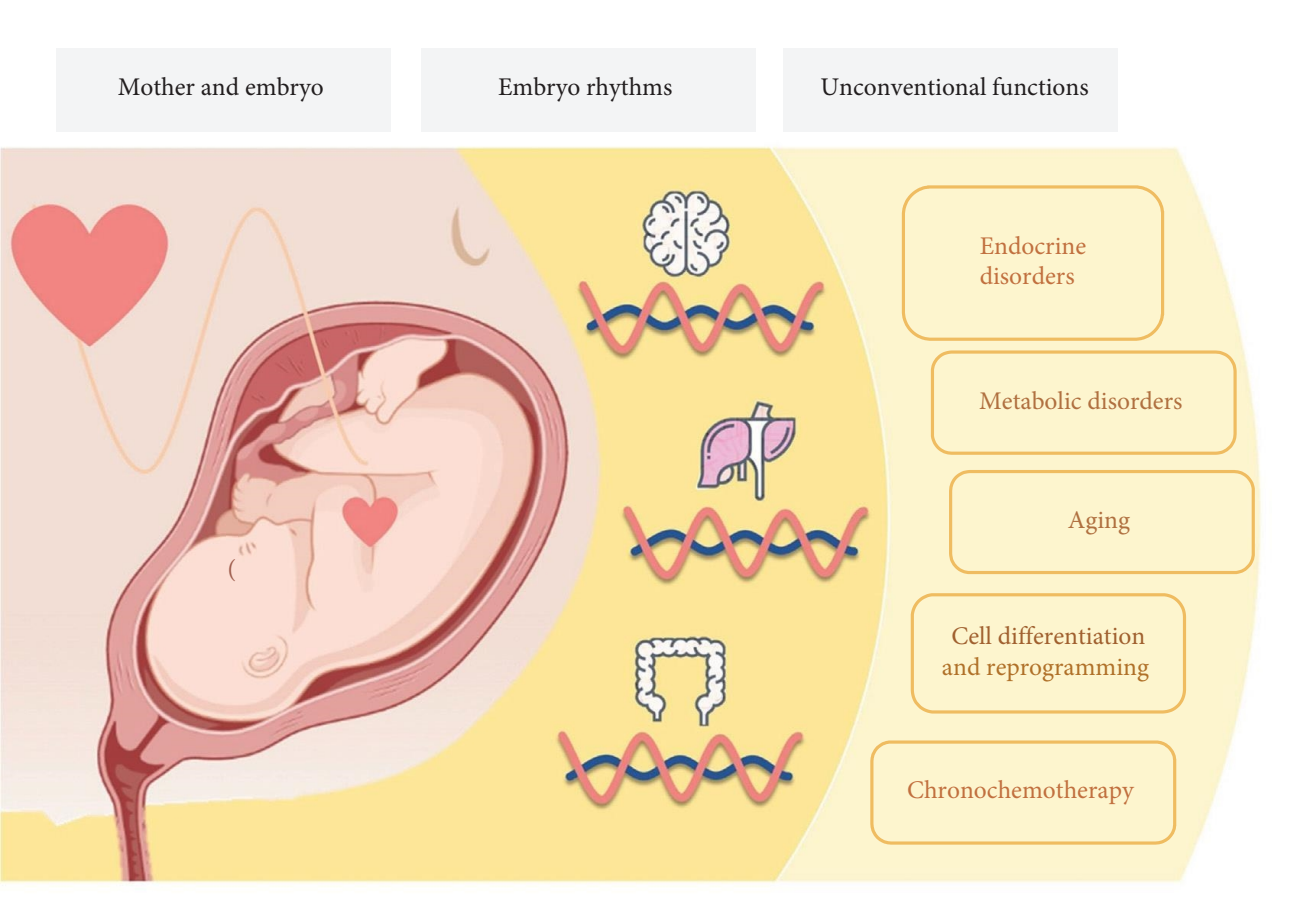
Circadian rhythms ensemble from embryos flow through life. Embryonic destiny is conceived in the mother, but it plays its own chapter of life from birth to death. Development of the circadian rhythm commences within the embryo, and is orchestrated by the harmonious interaction of both central and peripheral systems. Together, they form a symphony of life. Understanding the relationship between the circadian rhythm and the embryo development is imperative in advancing the field of chronobiology. By regulating circadian genes at various stages of embryonic development and differentiation, medicine may exploit the promising potential of chronobiology. Therefore, future research should focus on exploring the interplay between circadian rhythms and embryogenesis, to determine how this may be applied to improve health outcomes.

In this review of the literature, we have summarized a detailed account of the association between embryo rhythms and the maternal system, highlighted the various developmental milestones of embryo rhythms, and outlined the challenges and limitations of current research (Figure 1). We contend that during the period when the embryo rhythm has not yet matured to function independently, robust maternal rhythms are likely to conduct embryo rhythms before the generation of self-sustaining rhythm oscillations in the SCN, liver, kidney, adrenal gland, and intestinal systems, at various embryo dates, for the life ensemble. It is indisputable that circadian rhythms play an essential role in the process of embryo development, regulating maturation, and functional formation, and have immense potential to guide the field of research on stem cell differentiation and regenerative medicine. The combined action of circadian genes and circadian rhythm regulators promote rhythm growth in the embryo and continues postnatally, providing the strongest “pulse” of life. This symphonic coalescence of cell fate, orchestrated by circadian rhythms during embryo development, reflects the adaptation of mammals to the environment, their own evolution, and the synergistic coexistence between humanity and their environment.

## Figures and Tables

**Table 1:** Summary of initial oscillation in murine embryos.

Embryonic organs	Initial oscillation at	Circadian *genes*/proteins	References
Adrenal gland	ED18	*Bmal1*, *Per2*, Egr-1, Mt1, and StAR	[35]

Colon	ED20	*Bmal1*, *Per2*, and *Rev-erb*	[36]

Kidney	ED20	*CLOCK*, *Per2*, *Rev-erb*, aENaC, SGK1, NHE3, and AVPR2	[37]
	ED13	*Per2*	[7]
	ED18	—	[38]

Liver	ED13	*Per2*	[7]

Heart	ED17	*CLOCK*, *Bmal1*, *Cry1*, *Per2*, *Per3*, *Nr1d2* (*Rev-erbβ*), *and Dbp*	[25]

Pars tuberalis	ED18	*Cry1*, *Cry2*, *Per1*, and *Per2*	[39]

SCN	ED13	*Per2*	[7]
	ED14.5	*Per2*	[40]
	ED18	*Per1*, *Per2*, and AVP	[39]

*Note:* Bmal1, brain-muscle-arnt-like protein 1; Egr-1, early growth response protein 1; Cry1, cryptochrome circadian regulator 1; Cry2, cryptochrome circadian regulator 2; aENaC: epithelial sodium channel; Mt1, melatonin receptor; NHE3, sodium-hydrogen exchanger 3; Per1, circadian period regulator 1; Per2, circadian period regulator 2; Rev-erb, nuclear receptor subfamily 1 group DSGK1, serine/threonine protein kinase 1; StAR, acute regulatory protein.

Abbreviations: AVP, arginine vasopressin; Avpr2, arginine vasopressin receptor 2; CLOCK, circadian locomotor output cycles kaput; ED, embryonic day; SCN, suprachiasmatic nucleus.

## Data Availability

All data are available in the manuscript.
